# Discussion to: Concomitant electromagnetic navigation transbronchial microwave ablation of multiple lung nodules is safe, time-saving, and cost-effective

**DOI:** 10.1016/j.xjtc.2023.08.017

**Published:** 2023-08-22

See Article page 265.

Presenter: Dr Joyce W. Y. Chan

**Unidentified Speaker 1**. This abstract will be discussed by Dr Michael Lanuti from Massachusetts General Hospital.

**Dr Michael Lanuti***(Boston, Mass)*. Thank you. Thank you for the opportunity to discuss the paper and thank you, Dr Chan and your colleagues for sort of expanding this emerging technology more than anybody else. This particular catheter that you're using is not Food and Drug Administration-approved in the United States, this microwave catheter. But we're all sort of waiting on our heels, if you will, to use the technology. So, with that being said, this concept of studying bronchoscopic delivery of microwave thermal ablation to multiple nodules and many of us are just trying to get started on our first, is a great contribution to this emerging technology. And thank you for providing the manuscript in advance.

**Figure:**
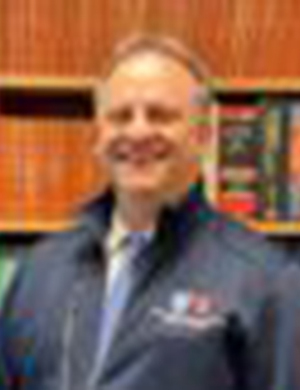

So, this modality of transbronchial delivery of thermal ablation has broad appeal. And it's sort of a hot topic for all of us. And many of us want to sort of jump in the arena, and we want to learn. And we're learning from essentially the folks that do it the most, which are mostly in this room. And so, the fact is that we're competing with pulmonologists who have access to the same navigation techniques that we do. So they're adopting the strategy very quickly as well. So, I would encourage our thoracic colleagues to be part of this emerging technology, only because we can treat the complications better than our pulmonary colleagues. So, you've been at the forefront, as I mentioned, of this. And what I would note is that the follow-up here is short. And that the pneumothorax rate is quite low. I imagine that those were pneumothoraxes that didn't get chest tubes, but we'll talk about that. So, the strength of this manuscript is that it actually gives you experience with all sorts of lessons learned. I think that's valuable to thoracic surgeons. So, I have 3 questions for you. And we'll do 1 at a time. What nodule characteristics or location would make a target not amenable to this strategy? Not so much the bronchus sign, but what are the things—location and impact, solid versus subsolid?

**Dr Joyce W. Y. Chan***(Hong Kong SAR)*. Thank you very much for your question. So, during our experience, we have tried numerous lesion locations, and we have found that the relative contraindication for this sort of technology is the medial one-third of the lung because they are too close to the very big vessels, which would cause a significant heat sink effect where the flowing blood in the large blood vessels would carry away much of the thermal energy. And also, we find that it is quite difficult to puncture through the relatively stronger bronchus at that area. In addition, we would also try to avoid ablating any of the important neurovascular structures. For example, the phrenic nerve, if the lesion is in the lingula, very close to the phrenic nerve, or high in the apex close to the sympathetic trunk, which may cause Horner's syndrome, then we would avoid those. But otherwise, lesions, for example, those shielded by the scapula, which would otherwise be less possible for percutaneous ablation, or those at the very base of the lung, we are able to reach those and ablate safely.

**Figure:**
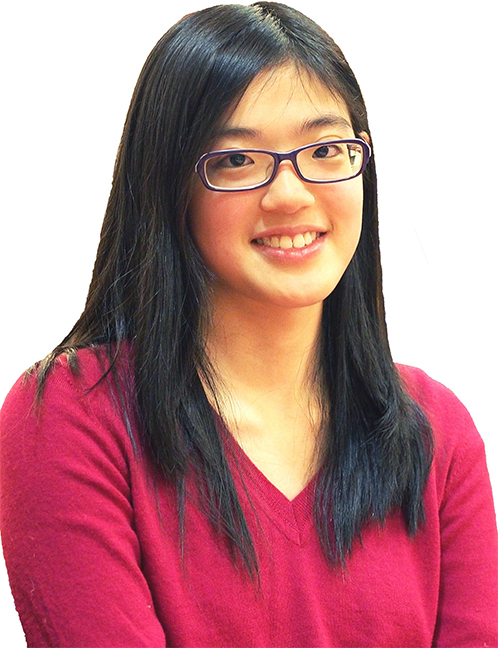

**Dr Lanuti**. Thank you. You mentioned that you observed an ablation margin of about 6 mm. When we do transthoracic thermal ablation, we generally try to reach 10 mm. Can you tell us, is there a limitation of the delivery or have you lowered your standard for margin?

**Dr Chan**. Oh, that's a very important question regarding margin. So, the way we measure the margin, as we've shown just now, is that we look at the ground-glass opacity, which represents the actual ablation zone, and measure it to the closest distance to the nodule edge. But what we observe is that there is significant contraction during ablation, which means that the lung nodule that is—the lung volume that is being ablated has some desiccation, and the nearby blood vessels get drawn into the center of ablation. So, taking that into account, the crude margin of 6.1 mm might in fact be well more than 1 cm in our series.

**Dr Lanuti**. And that's measured by cone beam at the time?

**Dr Chan**. Yes. At 10 minutes after the ablation.

**Dr Lanuti**. Okay. And then lastly, for those of us who are trying to use this emerging technology, how at your institution do you decide to treat patients? Do you have a multidisciplinary clinic where you have all the players in the room? I know that at our institution, which you'll hear in the next abstract, we now have an actual dedicated ablation clinic where we review every case. How do you guys manage?

**Dr Chan**. We do have a thoracic and multidisciplinary team involving the radiologist, oncologist, and also the pulmonologist. And we go case by case, and we take into account which nodules would be better for transbronchial ablation. For example, those that have higher risk of pneumothorax, those patients who have emphysema, or the nodule is very close to the pleura. And also, if there are multiple nodules in the lung that require ablation, then usually transbronchial ablation is preferable to the percutaneous approach.

**Dr Lanuti**. Thank you very much.

**Unidentified Speaker 2**. I really echo what Dr Lanuti said: This is a great effort by thoracic surgery and we're very proud of this. I noticed that you are ablating concomitant nodules, and I noticed on some of the images you showed that they were pure ground-glass opacities. Did you obtain pathologic confirmation of the diagnosis on each one of them? And when do you always treat concomitant ground-glass nodules? Every time, or do you sometimes only focus on a dominant nodule and leave the others?

**Dr Chan**. That's a very critical question. So, in our series; for example, these 56 nodules, around 8 of them had a definite pathological diagnosis of cancer. The rest of them are highly radiologically suspicious. For example, a patient with similar solid nodules in a patient who had previous lung resection for metastasis. And we do try to get a biopsy, but you can see that our main nodule size is only 9.3 mm. So sometimes biopsies are quite difficult. And a lot of them had prior biopsy results showing things like atypia, which are suspicious enough. And also, we don't routinely ablate all of these ground-glass opacities. We observe them first. And if there are any that enlarge in size or increase in solidity, then we would choose them to ablate.

**Unidentified Speaker 1**. One quick question.

**Unidentified Speaker 3**. I was, uh, surprised about the time of the procedure of a single nodule: 2 and a half hours. What are the rate-limiting steps? Because the microwave is usually quicker, and you just burn for 10 minutes or whatever the predetermined time is. What adds to that time to make it that duration.

**Dr Chan**. I think mostly it's the nodule selection. As I mentioned, bronchus sign was not present in 70% of our patients. In that case, we would need to use some transbronchial access tools. And sometimes it is a bit difficult to reach those very small lesions accurately. So, we took some time to navigate to the nodules. And we make sure that our margins are enough. Recently, we are also having more double ablations in the same region because we want to get a more definite margin. And that further increases the operative time.

**Unidentified Speaker 1**. Okay. Thank you very much for your presentation.

## Conflict of Interest Statement

Dr Ng is a consultant for Johnson and Johnson, Medtronic USA, and Siemens Healthineer and Dr Lanuti is a consultant for AstraZeneca. The other author reported no conflicts of interest.

The *Journal* policy requires editors and reviewers to disclose conflicts of interest and to decline handling or reviewing manuscripts for which they may have a conflict of interest. The editors and reviewers of this article have no conflicts of interest.

